# Unusual Bilateral Upper Extremity Pitting Edema in a Patient With Severe Dermatomyositis

**DOI:** 10.7759/cureus.15445

**Published:** 2021-06-04

**Authors:** Srikanth Mukkera, Anusha Ammu, Sudhir Bare, Lakshmi P Alahari, Srikanth Naramala

**Affiliations:** 1 Rheumatology, Texas Tech University Health Sciences Center at Permian Basin, Odessa, USA; 2 Internal Medicine, Texas Tech University Health Sciences Center at Permian Basin, Odessa, USA; 3 Hospital Medicine, Medical Center Hospital, Odessa, USA; 4 Rheumatology, Adventist Medical Center, Hanford, USA

**Keywords:** dermatomyositis, pitting edema, azathioprine, methotrexate, prednisone, tpmt, gottrons, combination, therapy, nxp

## Abstract

A 56-year-old Hispanic female presented with six weeks of progressive dysphagia, proximal muscle weakness, erythematous rash, bilateral upper extremity pitting edema, and left lower extremity pitting edema. She had preserved heart function and a normal echocardiogram (ECG). She presented with elevated creatine kinase (CK) and aldolase, with normal renal function. Muscle biopsy suggested idiopathic polymyositis. No blood clot was seen on deep vein thrombosis (DVT) ultrasound. The myositis antibody panel showed the NXP-2 antibody, which is usually seen in pediatric dermatomyositis cases.

In our literature search, extremity pitting edema is an unusual way of presentation in dermatomyositis. She responded with intravenous immunoglobulin (IVIg) and high-dose intravenous steroids. We used azathioprine for remission maintenance; her rash recurred after tapering steroids. We resumed tapering steroid therapy and started her on weekly methotrexate along with daily azathioprine. With this combination therapy, her rash and muscle function improved. We successfully tapered her steroids. In our literature search, combination therapy with azathioprine and methotrexate was not reported. Our patient is tolerating this therapy very well.

## Introduction

Dermatomyositis patients present with proximal muscle weakness, dysphagia, Gottron’s papule, heliotropic rash, shawl sign, and diffuse maculopapular rashes. Pitting edema is an unusual presentation of dermatomyositis [1]. Our patient presented with bilateral upper extremity, left lower extremity pitting edema, and bilateral knee cap Gottron’s papule.

The patient's muscle biopsy confirmed a diagnosis of dermatomyositis. She has no history of cancer, recent extensive cancer screening negative.

Her myositis antibody panel showed positive for nuclear matrix protein (NXP-2) antibody, which is also known as anti-MJ antibodies. These antibodies are not common in adults (1.6%) but are more commonly seen in juvenile dermatomyositis 25% [2].

Our patient lost around 30 pounds in one month due to severe dysphagia. She required a gastric feeding tube. She responded initially with intravenous immunoglobulin (IVIg) and high-dose pulse IV steroid therapy. We used azathioprine for the maintenance of remission. Her skin symptoms persisted despite the use of azathioprine. We added a low dose of methotrexate (10 mg PO) weekly to control her skin findings. The combination of azathioprine and low-dose methotrexate for the maintenance of dermatomyositis remission has not been studied extensively.

## Case presentation

**Figure 1:**
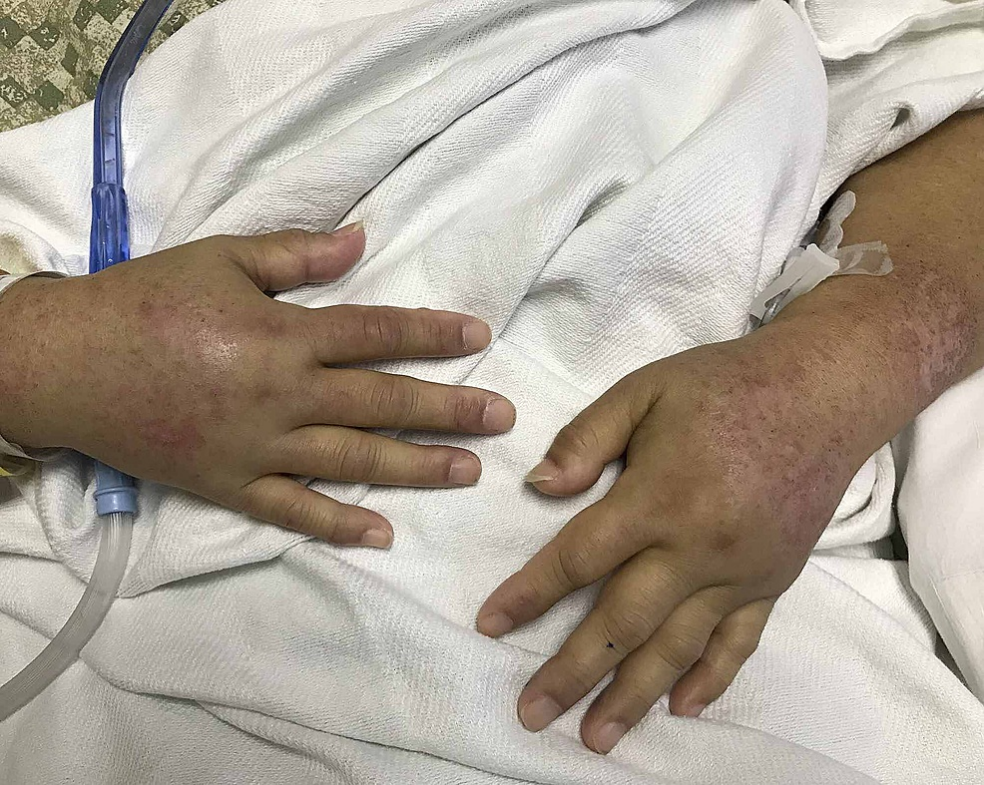
Bilateral upper extremity pitting edema and poikiloderma rash

A 56-year-old female with a past medical history of type 2 diabetes mellitus (with glycated hemoglobin (Hba1c) 6.8), hypertension, and hyperlipidemia was initially seen in a primary care setting for progressive worsening of facial swelling, urticarial rash, and bilateral upper extremities swelling six weeks prior to admission as shown in Figure 1. She was treated with EpiPen, steroids, and antihistamines considering it as an allergic reaction with angioedema. Workup at the office showed negative allergy testing. When symptoms worsened, the patient was admitted to the inpatient setting and further workup was pursued. Blood work showed elevated antinuclear antibody (ANA) levels (1:1280), speckled and homogeneous pattern, and positive ribonucleoprotein (RNP) antibody. Complements 3 and 4 were normal. Sjogren's, anti-Smith antibodies, scleroderma antibodies, and Jo-1 antibodies were negative.

Urinalysis did not show any hematuria, pyuria, and proteinuria. CRP was normal and antineutrophilic cytoplasmic antibody (ANCA) levels and glomerular basement membrane antibodies were negative. The initial working diagnosis was mixed connective tissue disease (MCTD)/lupus. The patient was sent home on hydroxychloroquine 200 mg daily and prednisone 30 mg daily.

She complained of dysphagia during her outpatient follow-up with the rheumatologist two weeks post-discharge. She was prescribed fluconazole and nystatin mouthwash, suspecting candidal esophagitis; prednisone was tapered down to 5 mg daily, and Plaquenil was continued. She went to the emergency room (ER) two weeks later with worsening dysphagia, unable to swallow her own saliva, 20-pound weight loss, and worsening bilateral upper extremity swelling with decreased range of motion.

**Figure 2:**
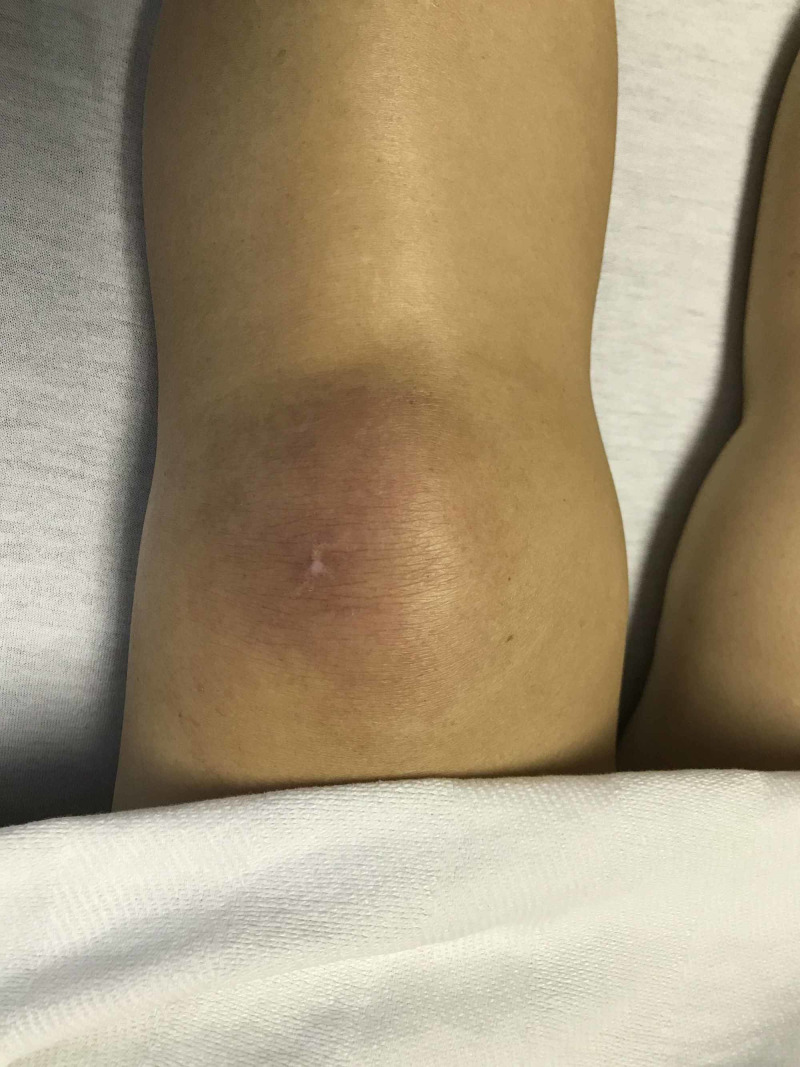
Left knee Gottron's papule

Vital signs in the ER were temperature 36.5 °Celsius, heart rate 72/min, respiratory rate 15, and blood pressure 109/63 mmHg. On examination, bilateral upper extremities were swollen and tender. She was not able to raise the arm above her head and was not able to stand up. The left lower extremity was grossly swollen with an erythematous rash on the knee as shown in Figure 2.

MRI of the brain was negative for acute infarct or bleeding. On Day 2 of admission, the patient underwent upper gastrointestinal endoscopy, which was negative for esophageal candidiasis, strictures, or obstruction. Speech pathology performed a barium swallow evaluation, which demonstrated profound swallow dysfunction with severe aspiration even to saliva. On Day 5 of hospitalization, the patient underwent percutaneous endoscopic gastrostomy (PEG) tube placement. Computed tomography of the thorax was negative for superior vena cava syndrome and showed normal lungs. Bilateral upper extremities Doppler ultrasound was negative for deep vein thrombosis.

On Day 7 of hospitalization, she developed a maculopapular rash on the bilateral forearm, hands, bilateral ears, bridge of the nose, and upper back. Blood work at that time showed elevated liver enzymes, Creatine kinase (CK) was elevated at 386 U/L, and aldolase was elevated at 11.3 U/L. MRI of bilateral upper extremities was consistent with myositis while MRI of the left lower extremity was also consistent with inflammatory myopathy involving multiple muscle groups. Based on the clinical presentation and history, inflammatory dermatomyositis was suspected. On Day 7, the patient was started on intravenous (IV) methylprednisolone 1 gram per day for three days and IV immunoglobulins for four days (total dose 2 gram/kg). On Day 8, the patient underwent a muscle biopsy of the left thigh. Further workup was negative for hepatitis panel, QuantiFERON-TB, cancer antigen (CA 15), CA 125, and carcinoembryonic antigen (CEA). The echocardiogram was normal.

Skeletal muscle biopsy of the left thigh showed necrotizing myopathy with mild neuropathic changes and type II myofiber atrophy with increased lipid stores. The myositis antibody panel was highly positive for NXP-2 antibodies, and the PM-SCL antibody had low reactivity.

SSA 52 antibody, SSA 60 antibody immunoglobulin G (IgG), anti-Smith, RNP antibodies IgG, anti-Jo 1 antibody, PL 12, PL 7, EJ antibody, OJ antibody, SRP antibodies, and Ku antibody were negative. Mi-2 antibodies, PT 155/140 antibodies, TIF 1 antibodies, ACE 1 antibodies, and MDA 5 antibodies were negative.

After treatment, the swelling in her bilateral upper extremities decreased, she gained strength, and she was able to ambulate. During her stay in the hospital, she was on IV Solu-Medrol 80 mg daily. She was discharged on prednisone 50 mg daily and azathioprine 100 mg daily.

She was sent home on oral prednisone taper (with a taper plan for 5 mg down every two weeks) along with azathioprine 100 mg daily. Her thiopurine methyltransferase test was normal. Her muscle power improved and CK and aldolase normalized.

Her skin rash recurred when she was on 20 mg prednisone. We added low-dose methotrexate 10 mg weekly, with which her skin symptoms resolved. She is currently on a combination of azathioprine 100 mg daily, methotrexate 10 mg oral weekly, folic acid 1 mg daily, and hydroxychloroquine 200 mg daily. Her prednisone was tapered down, and she is currently on 5 mg daily. In her case, MCTD is also a consideration, given the positive RNP and positive PM-SCL antibody. We monitored her labs frequently because of a combination of azathioprine and methotrexate therapy. She is tolerating this therapy without any cytopenias. It took five months to slowly taper her prednisone. Her feeding tube was removed after four months, as her swallowing difficulty resolved.

## Discussion

Dermatomyositis (DM) is an inflammatory myopathy (IIM) characterized by distinct skin lesions and a clinically wide range of systemic manifestations. The estimated prevalence rate is 1 to 6 per 100,000 adults in the United States. DM affects both genders with a 2:1 female: male ratio; it affects all ethnic groups but is more common in African Americans. It has a bimodal distribution, with juvenile DM (JDM) diagnosed between ages four and 14 and adult DM diagnosed between 40 and 60 years of age [2].

The pathogenesis of DM is multifactorial. It is triggered by environmental factors along with underlying genetic factors resulting in modulation of immune mechanisms and activation of an auto-inflammatory cascade. Known environmental triggers are ultraviolet radiation, viral infections, medications, smoking, tattoo, and ingestion of herbal supplements [3-4]. Multiple studies showed an association between DM and MHC complex, especially the HLA-DRB1 allele [5]. The immune mechanisms involved in pathogenesis are antibody-mediated complement cascade activation, cytotoxic CD8 + T-cells pathway initiation, and clonal B cell proliferation resulting in autoantibody production [6-7]. In addition, there is also evidence of the upregulation of the interferon pathway and tumor necrosis factor (TNF) alpha expression on the muscle, skin, and blood of DM patients correlating with disease activity [8]. Mediators of the innate immune system, such as toll-like receptors, are also expressed on the sarcolemma of muscle fibers in biopsy specimens of myositis [9]. Apart from inflammatory pathways, non-immune mechanisms like ER-stress, NFκB-activation, and free radicals, such as nitric oxide, are also noted to contribute to the weakness of the skeletal muscle in the absence of structural damage [10]

DM has several subsets of variants such as classical DM, clinically amyopathic DM (CADM), hypomyopathic DM, postmyopathic DM, and adermatopathic DM (11). Adermatopathic DM presents as weakness and has histological signs similar to DM but without inflammatory skin lesions. CADM has only skin manifestations but no weakness of the muscles. It often affects the lungs in the form of interstitial lung disease (ILD) [11]. Patients with DM present as painless acute or subacute onset of symmetric, proximal muscle weakness, pathognomonic skin lesions, Gottron papules, heliotrope rash, shawl sign, V sign, and Holster sign. Nailfold changes that are usually seen are periungual erythema, telangiectasias, and hemorrhagic nail fold infarcts. Scalp involvement is sometimes seen with atrophic, erythematous, or pruritic scaly plaques. Cutaneous vasculitis, Calcinosis cutie, and mechanic's hands are rare. Systemic organs get affected sometimes especially the gastrointestinal tract, lungs, heart, joints, and kidneys [12]. Pulmonary manifestations include pulmonary hypertension, serositis, and various degrees of Interstitial lung disease. DM presenting with subcutaneous non-pitting edema is a rare manifestation. Microinfarction was found to be the predominant underlying pathology [13].

Biopsy specimens of muscle show perifascicular atrophy with inflammatory infiltrates like macrophages, CD20+ B cells, CD4+ T cells, CD25+ plasma cells, and plasmacytoid dendritic cells in perimysial and perivascular regions. There is also an increased perifascicular expression of MHC class I. But because of patchy involvement, false negatives are seen. Muscle enzymes, such as serum creatine kinase (CK), levels are 10-50 fold elevated. DM-specific antibodies like anti-Mi2, anti-melanoma differentiation-associated protein 5 (MDA5), anti-NXP2, anti-TIF1, and anti-small ubiquitin-like modifier activating enzyme (SAE) have been identified in recent years [14].

NXP-2 is a protein that helps in the regulation of nuclear transcription essential for the activation of tumor suppressor gene p53 and ribonucleic acid (RNA) metabolism. Anti-NXP2 antibodies (Abs) suppress the function of these regulatory proteins, disturb intracellular processes and the mechanism of apoptosis, and result in acquiring malignant potential in some cells. The NXP-2 Abs, originally termed anti-MJ, are uncommon in adults (1.6% of patients). Conversely, they are more frequently seen in juvenile dermatomyositis, affecting 15%-25% of children. They present with severe myopathy, atrophy, contracture development, and significant compromise of the functional status secondary to vasculopathy-induced muscle ischemia. The cutaneous hallmark in this subset is the development of calcinosis cutis, subcutaneous edema, and distal ulcerations. These individuals are more prone to vasculopathy-induced gastrointestinal bleeding. They are also associated with an increased risk of underlying malignancy and poor prognosis.

Pulse dose glucocorticosteroids are the mainstay of the treatment, particularly in acute and severe cases. The treatment can be initiated with an IV high-dose pulse of 250-1000 mg per day for three to five days. Steroid tapering should not be done solely based on CK values, but clinical improvement based on the Medical Research Council (MRC) sum score needs to be monitored [15]. Long-term immunosuppression with either methotrexate (MTX), azathioprine (AZA), or mycophenolate mofetil (MMF) should be started along with the steroid [16]. Other alternative options like IV rituximab, oral cyclosporine, or intravenous immunoglobulin G (IVIg) can be considered if a standard regimen with steroids and immunosuppressants is not sufficiently effective [17-18]. Skin lesions require protection from sunlight/UV exposure; add-on treatment with topical glucocorticosteroids and IVIG has been shown efficacious. The management of ILD requires an interdisciplinary approach with a pulmonologist and requires more aggressive treatment with a combination of high-dose glucocorticosteroids and immunosuppressants such as AZA plus either RTX or CYC [19].

Patients with dermatomyositis should undergo comprehensive malignancy screening rather than a focused or a specific cancer screening. Adenocarcinoma of lung, pancreas, gynecological cancers, including ovarian and cervix; gastrointestinal, including stomach and pancreas; and bladder cancer account for approximately 70% of the patients presenting with dermatomyositis or polymyositis. Treatment of the tumor should be prioritized vs. immunosuppression.

## Conclusions

Dermatomyositis presenting with bilateral upper extremity pitting edema is rare. A review of the literature reported only a few cases with upper extremity pitting edema. First-line conventional therapy for remission maintenance is either methotrexate or azathioprine. A combination of azathioprine with methotrexate is not studied extensively. In our patient, muscle weakness improved with prednisone and azathioprine 100 mg daily. We tapered her prednisone 5 mg down every two weeks. It took five months to reach prednisone 5 mg daily. Her skin symptoms with rash and pitting edema recurred when we reached prednisone dose 20 mg daily. At this time, we continued azathioprine 100 mg daily and added low-dose methotrexate 10 mg oral weekly and folic acid 1 mg daily. With this combination of therapy, we managed to control her muscle weakness and skin findings effectively. Her pitting edema also resolved.
